# *QuickStats:* Age-Adjusted Percentage* of Adults Aged ≥18 Years Who Were Never in Pain, in Pain Some Days, or in Pain Most Days or Every Day in the Past 6 Months,^†^ **by Employment Status**^§^ **— National Health Interview Survey,**^¶^ **United States, 2016**

**DOI:** 10.15585/mmwr.mm6629a8

**Published:** 2017-07-28

**Figure:**
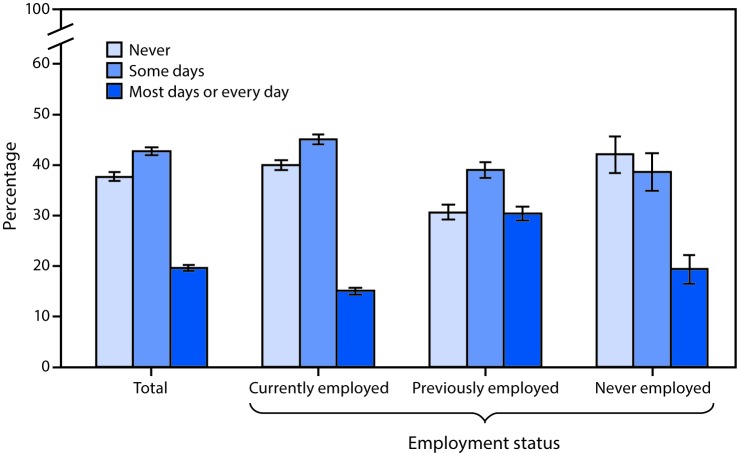
In 2016, 37.7% of adults aged ≥18 years never had pain, 42.8% had pain on some days, and 19.6% had pain most days or every day in the past 6 months. A higher percentage of adults who were previously employed (30.4%) had pain most days or every day compared with never employed adults (19.4%) and currently employed adults (15.1%). Never employed adults (42.0%) and currently employed adults (39.9%) were more likely to report never having had pain than previously employed adults (30.7%).

**Source:** National Health Interview Survey, 2016. https://www.cdc.gov/nchs/nhis.htm.

