# *Erratum:* Vol. 66, No. 29

**DOI:** 10.15585/mmwr.mm6644a10

**Published:** 2017-11-10

In “*QuickStats*: Age-Adjusted Percentage of Adults Aged ≥18 Years Who Were Never in Pain, in Pain Some Days, or in Pain Most Days or Every Day in the Past 6 Months, by Employment Status — National Health Interview Survey, United States, 2016,” on page 796, the caption should have read as follows:

“In 2016, **38.1%** of adults aged ≥18 years never had pain, **42.6%** had pain on some days, and **19.4%** had pain most days or every day in the past 6 months. A higher percentage of adults who were previously employed (**29.8%**) had pain most days or every day compared with never employed adults (**18.6%**) and currently employed adults (15.1%). Never employed adults (**41.9%**) and currently employed adults (**40.4%**) were more likely to report never having had pain than previously employed adults (**31.2%**).”

